# TSG (2,3,4’ ,5-tetrahydroxystilbene 2-O-β-D-glucoside) suppresses induction of pro-inflammatory factors by attenuating the binding activity of nuclear factor-κB in microglia

**DOI:** 10.1186/1742-2094-10-129

**Published:** 2013-10-21

**Authors:** Chao Huang, Yuzhe Wang, Jia Wang, Wenjuan Yao, Xiangfan Chen, Wei Zhang

**Affiliations:** 1 Department of Pharmacology, School of Medicine, Nantong University, 19 Qixiu Road, Nantong 226001, Jiangsu, China

**Keywords:** 2,3,4’,5-tetrahydroxystilbene 2-O-β-D-glucoside, Pro-inflammatory factors, Nitric oxide, Inhibitor of κB-α, Nuclear factor-κB, Microglia, BV-2 cells

## Abstract

**Background:**

Induction of pro-inflammatory factors is one of the characteristics of microglia activation and can be regulated by numerous active components of Chinese traditional herbs. Suppression of pro-inflammatory factors is beneficial to alleviate microglia-mediated cell injury. The present study aims to investigate the effect and possible mechanism of 2,3,4’,5-tetrahydroxystilbene 2-O-β-D-glucoside (TSG) on LPS-mediated induction of pro-inflammatory factors in microglia.

**Methods:**

Western blot, ELISA, and Hoechst 33258 were used to measure the protein expression, TNF-α/IL-6 content, and apoptotic nuclei, respectively. The mRNA level was measured by real time-PCR. Nitric oxide (NO) content, lactate dehydrogenase (LDH) content, and NF-κB binding activity were assayed by commercial kits.

**Results:**

TSG reduced iNOS protein expression as well as TNF-α, IL-6, and NO content in LPS-stimulated BV-2 cells. TSG attenuated the increase in apoptotic nuclei, caspase-3 cleavage, and LDH content induced by BV-2 cell-derived conditioned medium in primary hippocampal neurons. Mechanistic studies showed that TSG reduced the mRNA level of iNOS, TNF-α, and IL-6. TSG failed to suppress IκB-α degradation, NF-κB phosphorylation and nuclear translocation, and ERK1/2, JNK, and p38 phosphorylation. TSG, however, markedly reduced the binding of NF-κB to its DNA element. Chromatin immunoprecipitation (ChIP) assays confirmed that TSG reduced NF-κB binding to the iNOS promoter. These findings were ascertained in primary microglia where the LPS-induced increase in iNOS expression, NO content, apoptotic nuclei, and NF-κB binding to its DNA element were diminished by TSG.

**Conclusions:**

These studies demonstrate that TSG attenuates LPS-mediated induction of pro-inflammatory factors in microglia through reducing the binding activity of NF-κB. This might help us to further understand the pharmacological role of TSG in inflammatory response in the central nervous system.

## Background

Microglia are the resident immune cells in the central nervous system (CNS). They serve as the neuron-pathological sensor under various conditions such as inflammation [1,2]. The sensing of microglia to pathological stimuli leads to activation of microglia, which then produce trophic factors that are important for neuronal recovery [3]. However, uncontrolled activation of microglia triggers neurotoxicity by overproducing cytokines such as nitric oxide (NO), TNF-α, and IL-6 [4-6]. NO is synthesized by a family of NO synthase (NOS) consisting of three isoforms: endothelial NOS (eNOS), neuronal NOS (nNOS), and inducible NOS (iNOS) [7]. NO generated from eNOS and nNOS primarily participates in cardiovascular regulation, neuronal signaling transduction, and neuronal protection [8-10]. NO produced from iNOS [11], however, promotes the development of neurodegenerative disorders associated with inflammation such as Parkinson’s disease and multiple sclerosis [12,13]. Similarly, TNF-α and IL-6 also have dual functions. For example, TNF-α preconditioning was found to protect neurons from Aβ-mediated cell toxicity [14]. IL-6 treatment ameliorates trimethyltin-induced injury in neurons [15]. Both TNF-α and IL-6 were confirmed to enhance neurotoxicity [5,6]. Collectively, control of induction of cellular cytokines in microglia might be important for regulation of numerous physiological or pathological processes.

An important feature of pro-inflammatory factors is their absence in quiescent inflammatory cells and induction by inflammatory inducers. Lipopolysaccharide (LPS) is an extensively characterized inducer of pro-inflammatory factors [16]. It stimulates gene transcription of pro-inflammatory factors through the classical inhibitor of κB kinase (IKK)-inhibitor of κB α (IκB-α)-nuclear factor κB (NF-κB) signaling pathway. LPS binds with the Toll-like receptors leading to IκB-α degradation through the ubiquitin-proteasome system [17]. The removal of IκB-α liberates transcriptional factor NF-κB. The active NF-κB is then free for translocation to the nucleus, where it initiates gene transcription [17]. Besides IκB-α-NF-κB signals, mitogen-activated protein kinase (MAPK) including ERK1/2, p38, and JNK is also involved in induction of pro-inflammatory factors [18-20]. Interfering with the MAPK signals is beneficial to coping with inflammation.

Plenty of small molecules extracted from traditional Chinese herbal medicines have been reported to regulate induction of pro-inflammatory factors through the classical IκB-α-NF-κB pathway. For instance, oregonin was found to inhibit iNOS gene transcription by reducing the nuclear translocation of NF-κB in LPS-stimulated microglia [21]; curcumin suppresses the expression of NF-κB-dependent genes in rats [22]. A monomer of stilbene from a traditional Chinese herbal medicine polygonummultiflorum, 2,3,4’ ,5-tetrahydroxystilbene 2-O-β-D-glucoside (TSG), has also been found to attenuate inflammatory responses [23]. Its anti-inflammatory function was supported by the following evidence: 1) TSG suppresses COX-2 expression in a carrageenin-induced rat paw edema model [24]; 2) TSG reduces NO levels in serum and the aorta in atherosclerotic rats [25]; and 3) TSG decreases iNOS expression and infarct volume in the ischemic brain [26]. In the third case, the effect of TSG was explained by the suppression of NF-κB nuclear translocation in neurons but not in microglia [26]. In the present study, in view of the great importance of microglia in CNS disorders associated with inflammation [27,28], we explored the effect and mechanism of TSG on LPS-mediated inflammatory response in microglia. We found that TSG reduces iNOS expression and NO, TNF-α, and IL-6 release in microglia in a way that is independent of MAPK-IκB-α-NF-κB activation but likely represses NF-κB binding activity.

## Methods

### Chemicals and reagents

DMEM/F12 was obtained from Gibco Invitrogen Corporation (Carlsbad, CA, USA). Heat-inactivated FBS was purchased from Hyclone (Logan, UT, USA). TSG was the product of the National Institute for the Control of Pharmaceutical and Biological Products (Beijing, China). LPS, poly-L-lysine, and Hoechst 33258 were purchased from Sigma (Saint Louis, MO, USA). Antibodies against iNOS, IκB-α, p-NF-κB, NF-κB, Histone H2A, ERK1/2, phospho-ERK1/2, and glyceraldehyde-3-phosphate dehydrogenase (GAPDH) were purchased from Cell Signaling Technology (Beverly, MA, USA). Protein A/G PLUS-Agarose and antibodies against caspase-3, p38, phospho-p38, JNK, and phospho-JNK were the products of Santa Cruz Biotechnology (Santa Cruz, CA, USA). Other related agents were purchased from commercial suppliers. All drugs were prepared as stock solutions, and stock solutions were stored at -20°C.

### Cell preparation

BV-2 cells were grown in DMEM/F12 with 10% FBS. The use of mice was approved by the University Animal Ethics Committee of Nantong University (Permit Number: 2110836). Mouse primary cultured brain cells were prepared as described previously with some modifications [29]. Briefly, newborn (day 0 to 1) C57/BL6 mice were decapitated, hippocampus were then removed and digested with 0.125% trypsin for 15 minutes at 37°C. Followed by trituration and centrifugation at 118 *g* for 6 minutes, cells were re-suspended and plated on poly-L-lysine (1 mg/mL)-coated culture flasks. For preparation of hippocampal neurons, the single-cell suspension was cultured in DMEM/F12 supplement with 2% B_27_ and 1% penicillin-streptomycin (100 U/mL), and the medium was replaced every 3 days. For preparation of primary microglia, the individual cell suspensions were cultured in DMEM/F12 supplement with 10% FBS and 1% penicillin-streptomycin (100 U/mL). This medium was replaced every 3 days. After 12 days, mixed cells were shaken gently overnight (37°C, 18 h), and the supernatants were collected and plated on the new poly-L-lysine-coated culture flasks. All cells were maintained in a 37°C incubator containing 95% air and 5% CO_2_.

### Cell viability assay

Cell viability was measured using MTT Cell Proliferation and Cytotoxicity Assay Kit (Bi Yuntian Biological Technology Institution, Shanghai, China). Briefly, methylthiazolyldiphenyl-tetrazolium bromide (5 mg/mL) was dissolved in prepared MTT-dissolved solutions and kept at -20°C. After washing with PBS, the cells in plates were added 20 μL of MTT solutions and kept at 37°C for 4 h. The blue crystals were dissolved in formazan-dissolved solutions. The absorbance was read at 570 nm.

### Western blot

To extract the total proteins, cells were lysed on ice for 30 minutes in lyses buffer (50 mM Tris–HCl, pH 7.4, 1 mM EDTA, 100 mM NaCl, 20 mM NaF, 3 mM Na_3_VO_4_, 1 mM PMSF with 1% (v/v) Nonidet P-40, and protease inhibitor cocktail). The lysates were centrifuged at 12,000 *g* for 16 minutes, and the supernatants were harvested. After denaturation, 30 μg of protein was separated on 10% SDS/PAGE gels and then transferred to nitrocellulose membranes (Bio-Rad, Hercules, CA, USA). After blocking with 5% nonfat dried milk powder/Tris-buffered saline Tween-20 (TBST) for 1 h, membranes were probed with 1:500 primary antibodies against iNOS, caspase-3, IκB-α, p-NF-κB, NF-κB, ERK1/2, p-ERK1/2, JNK, p-JNK, p38, p-p38, and Histone H2A or 1:10,000 primary antibody against GAPDH overnight at 4°C. Primary antibodies were then removed by washing the membranes three times in TBST. Membranes were further incubated for 2 h at room temperature with IRDye 680-labeled secondary antibodies (1:3,000 to 1:5,000). Finally immunoblots were visualized by scanning using the Odyssey CLx western blot detection system. Isolated cytoplasmic and nuclear proteins were normalized to GAPDH and Histone-H2A respectively. The band density was quantified using Image J software.

### Real-time PCR

At the end of each treatment, total RNA was isolated from BV-2 cells using the RNeasy mini kit according to the manufacturer’s instructions (Qiagen, GmbH, Hilden, Germany). First-strand cDNA was generated by reverse transcription of total RNA using the RT system (Promega, Madison, WI, USA). Real-time PCR reactions were conducted with FaststartSYBR Green Master Mix (Roche Molecular Biochemicals, Shanghai, China). Briefly, 2 μL of diluted cDNA, 0.5 μM primers, 2 mM MgCl_2_, and 1 × FastStartSYBR Green Master mix were employed. The primers are quoted as follows [30,31]: iNOS 5’-CTC ACT GGGACAGCA CAG AA-3’ (forward), 5’-TGG TCA AAC TCT TGG GGT TC-3’ (reverse); TNF-α 5’-CTG TGA AGG GAA TGG GTG TT-3’ (forward), 5’-GGT CAC TGT CCC AGC ATC TT-3’ (reverse); IL-6 5’-TTC CAT CCA GTT GCC TTC TT-3’ (forward), 5’-CAG AAT TGC CAT TGC ACA AC-3’ (reverse); 18S rRNA 5’-GTA ACCCGTTGAACC CCA TT-3’ (forward), 5’-CCA TCCAATCGG TAG TAG CG-3’ (reverse). PCR products were detected by monitoring the fluorescence increase of double-stranded DNA-binding dye SYBR Green during amplification. The expression levels of target genes were normalized to the housekeeping gene (18S rRNA). The fold changes in the target gene expression between experimental groups were expressed as a ratio. Relative gene expression was calculated by the comparative cycle threshold (Ct) method. Melt-curve analysis and agarose gel electrophoresis were used to examine the authenticity of the PCR products.

### Detection of NO

Total nitrite levels in collected supernatants were measured with a Griess reagent kit (Invitrogen). The reaction consisted of 20 μL of Griess Reagent, 150 μL of supernatant, and 130 μL of de-ionized water. After incubation of the mixture for 30 minutes at room temperature, nitrite levels were measured at 548 nm using an M2 spectrophotometric microplate reader (Molecular Devices).

### Measuring of lactate dehydrogenase (LDH)

For LDH testing, hippocampal neurons were seeded into 24-well plates. BV-2 cells were treated with LPS (2 μg/mL) for 24 h, and then the supernatants were collected to incubate hippocampal neurons. After additional 48 h, the supernatants from hippocampal neurons were collected to be prepared for further experiments. LDH levels were determined using LDH Cytotoxicity Assay Kit according to the supplier’s recommendation (Bi Yuntian Biological Technology Institution, Shanghai, China).

### Hoechst 33258 staining

For Hoechst 33258 staining, hippocampal neurons in different groups were fixed with 2% paraformaldehyde in 0.01 M PBS (pH 7.4) for 20 minutes, and then rinsed three times with PBS for 10 minutes each. Cells were treated with Hoechst 33258 staining solution for 15 minutes at 4°C. The images of Hoechst 33258 staining were viewed with a Nikon Eclipse 800 microscope. Cells with condensed bright nuclei were regarded as apoptotic cells. The apoptosis rate was calculated by the ratio between the numbers of cells with condensed bright nuclei and total cell numbers. The numbers of apoptotic or total cells were counted from the resulting four phases for each point with the digital camera and microscope, and then averaged for each experimental condition. The data presented were generated from three separate assays.

### NF-κB binding assays

The nuclei were extracted from BV-2 cells or primary microglia by first incubating them in hypotonic buffer (10 mM Tris–HCl, pH 7.5, 10 mM NaCl, 1.5 mM MgCl_2_) at 4°C for 15 minutes. After homogenization, cell homogenates were spun at 3,000 *g* for 5 minutes. The supernatants were collected for western blot analysis. The pellets were recovered, extensively washed, and re-suspended in the nuclear extraction buffer (50 mM Tris–HCl, pH 7.4, 150 mM NaCl, 1% Nonidet P-40, 0.25% sodium deoxycholate, 10% glycerol, 50 mM NaF, 1 mM Na_3_VO_4_, 5 mM sodium pyrophosphate, protease inhibitors). The NF-κB binding activity of nuclear extracts was measured with the TransFactor NF-κB colorimetric kit (Clontech, Mountain View, CA, USA) according to the manufacturer’s instruction.

### Chromatin immunoprecipitation (ChIP)

The ChIP experiment was performed as described previously with some modifications [32]. Briefly, BV-2 cells were treated with LPS (2 μg/mL) for 1 h in the presence and absence of TSG. 1% of formaldehyde was added to the culture medium, and after incubation on the rocker for 10 minutes at room temperature, cells were rinsed twice with PBS and lysed for 15 minutes at 4°C. After sonication, the lysate was used as DNA input control. The remaining lysates were diluted 10-fold with ChIP dilution buffer followed by incubation with NF-κB p65 antibody overnight at 4°C. Immunoprecipitated complexes were collected using protein A/G Plus-agarose beads. The precipitates were extensively washed and then incubated in the elution buffer containing 1% SDS and 0.1 M NaHCO_3_ at room temperature for 20 minutes. Cross-linking of protein-DNA complexes was reversed at 65°C for 4 h. DNA was extracted with the QiagenPCR purification kit. For ChIP assays, we used the following primers (forward) 5’-CAAGCCAGGGTATGTGGTTT-3’ and (reverse) 5’-GCAGCAGCCATCAGGTATTT-3’ to get a fragment at 290-bp. The resulting product was separated by 2% agarose gel electrophoresis.

### Statistical analysis

Data are expressed as means ± standard error (SE). One-way analysis of variance (ANOVA) followed by the post hoc test was used for the statistical analysis, employing SPSS 11.0 software. Differences were considered significant at *P* < 0.05.

## Results

### TSG suppresses the induction of pro-inflammatory factors in LPS-stimulated BV-2 cells

**Figure 1:**
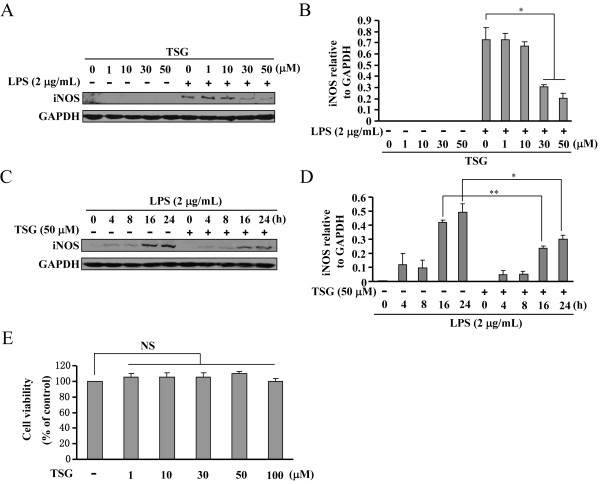
**Effects of TSG on induction of inducible nitric oxide (iNOS) protein in BV-2 cells stimulated with lipolysaccharide (LPS). (A)** Representative images showing that TSG pretreatment (30 minutes) inhibited iNOS expression in LPS-stimulated BV-2 cells at different concentrations (1, 10, 30, 50 μM). **(B)** Quantitative analysis of iNOS expression in cells upon LPS/TSG treatment (*P < 0.05 versus control). **(C)** Representative images showing the time-dependent effect of TSG on iNOS expression in LPS-stimulated BV-2 cells. **(D)** A time-course analysis of iNOS expression upon TSG incubation (*P < 0.05, **P < 0.01 versus control). **(E)** Quantitative analysis of the cell viability after TSG treatment at concentrations ranging from 1 to 50 μM (n = 6). All data were shown as mean ± standard error. Experiments were performed three times independently. NS, no significance; GAPDH, glyceraldehyde-3-phosphate dehydrogenase.

**Figure 2:**
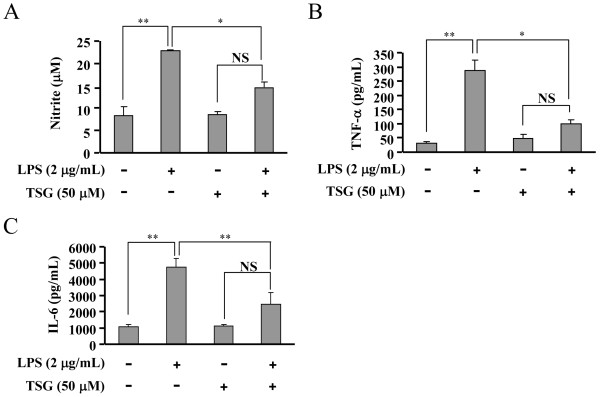
**Effects of TSG on release of nitric oxide (NO), TNF-α, and IL-6 in BV-2 cells stimulated with lipolysaccharide (LPS).** Quantitative analysis of NO **(A)**, TNF-α **(B)**, and IL-6 **(C)** content after TSG treatment (50 μM, 30 minutes) in LPS-stimulated BV-2 cells (n = 6, **P < 0.01 versus control; *P < 0.05 or **P < 0.01 versus the LPS alone-treated group). NS, no significance. All data were shown as mean ± standard error.

To determine the working concentration and effective period of TSG for induction of pro-inflammatory factors, we first investigated the dose- and time-dependent effects of TSG on iNOS expression in BV-2 cells. BV-2 cells were pretreated with TSG for 30 minutes at concentrations range at 1 to 50 μM when 2 μg/mL of LPS was applied to induce iNOS expression. As shown in Figure 1A and 1B, TSG significantly reduced the increase in iNOS expression in LPS-stimulated (2 μg/mL, 24 h) BV-2 cells. Peak inhibition was observed at the concentration of 50 μM. For this reason, 50 μM of TSG was selected for the following experiments. A time-dependent response curve showed that pretreatment of BV-2 cells with TSG (50 μM, 30 minutes) markedly inhibited the increase in iNOS expression at the time points of 16 and 24 h (Figure 1C and D). The cell viability of BV-2 cells was not affected by TSG administration at 1 to 100 μM (Figure 1E). Consistent with the effect on iNOS expression, production of NO was also decreased by TSG treatment (50 μM, 30 minutes) in LPS-stimulated BV-2 cells: the content of NO was decreased from 22.93 ± 0.19 to 14.89 ± 1.16 (n = 6, *P* < 0.05 versus the LPS alone-treated group, Figure 2A). Finally, we observed a considerable reduction in TNF-α (from 286.65 ± 38.55 to 100.54 ± 13.42, n = 6, *P* < 0.05 versus the LPS alone-treated group) and IL-6 (from 4763.40 ± 529.09 to 2479.38 ± 706.54, n = 6, *P* < 0.01 versus the LPS alone-treated group) content after TSG treatment (50 μM, 30 minutes) in LPS-stimulated BV-2 cells (Figure 2B and C).

### TSG prevents primary hippocampal neuron injury induced by BV-2 cell-derived conditioned medium

**Figure 3:**
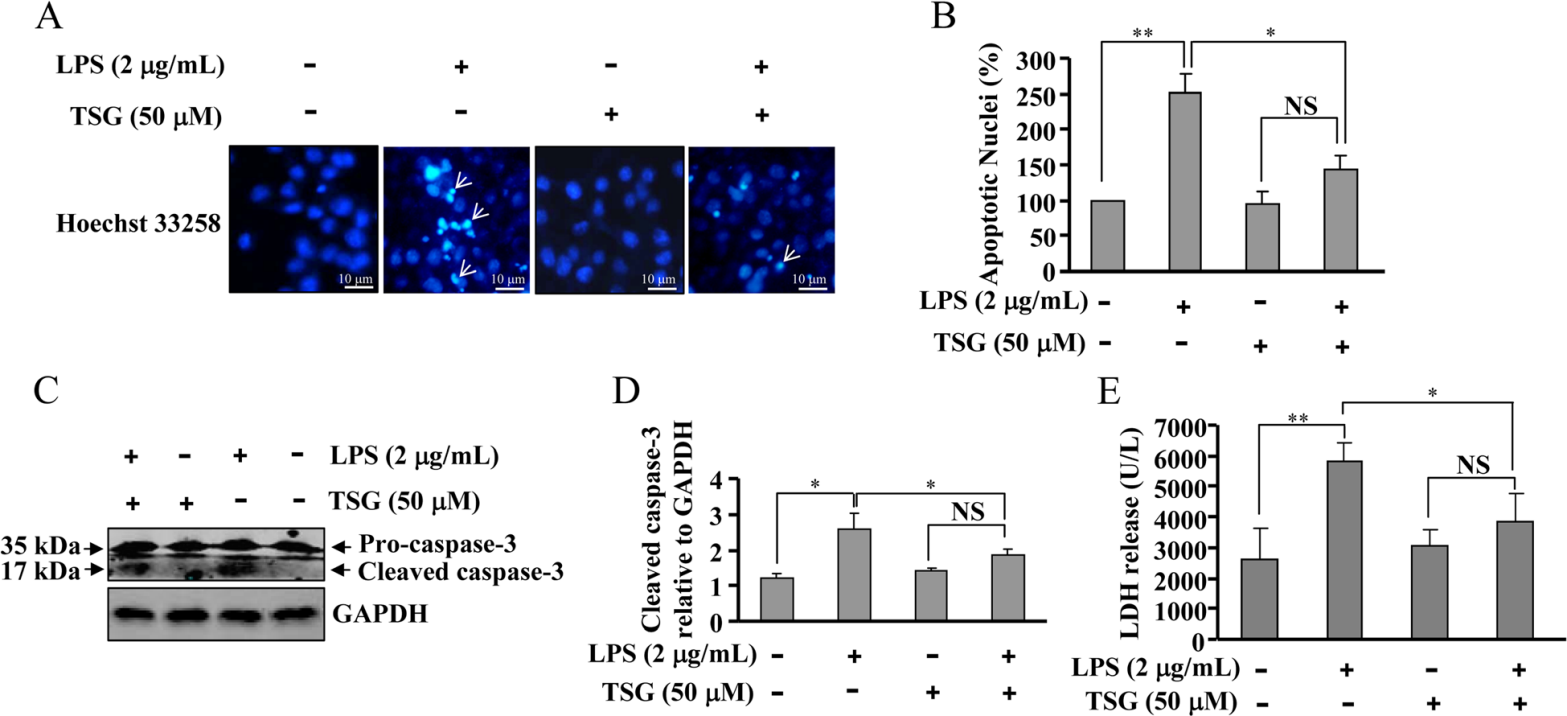
**Role of TSG in injury of hippocampal neurons induced by BV-2 cell-derived conditioned medium. (A)** Pretreatment with TSG (30 minutes, 50 μM) inhibited the increase in apoptotic nuclei in primary hippocampal neurons stimulated with BV-2 cell-derived conditioned medium. **(B)** Quantitative analysis of apoptotic nuclei percentage in neurons (n = 5, **P < 0.01 versus control; *P < 0.05 versus the lipopolysaccharide (LPS) alone-treated group). **(C)** Representative images showing that TSG pretreatment (50 μM, 30 minutes) reduced the cleavage of caspase-3 in primary hippocampal neurons stimulated with BV-2 cell-derived conditioned medium. **(D)** Quantitative analysis of cleaved caspase-3 in neurons (n = 3, *P < 0.05 versus control or the LPS alone-treated group). **(E)** Quantitative analysis of lactate dehydrogenase (LDH) content in LPS/TSG-treated hippocampal neurons (n = 6, **P < 0.01 versus control; *P < 0.05 versus the LPS alone-treated group). All data were shown as mean ± standard error. NS, no significance; GAPDH, glyceraldehyde-3-phosphate dehydrogenase.

To further investigate whether the TSG-mediated suppression of pro-inflammatory factors in BV-2 cells has protective roles in neuronal damage, primary hippocampal neurons were incubated with BV-2 cell-derived conditioned medium in the absence or presence of TSG. We found that without TSG treatment, the conditioned medium induced a marked increase in apoptotic nuclei percentage (Figure 3A and B), cleaved caspase-3 level (Figure 3C and D), and LDH content in primary hippocampal neurons (Figure 3E). After TSG treatment (50 μM, 48 h), the percentage of apoptotic nuclei, the level of cleaved caspase-3, and the content of LDH were decreased from 251.17 ± 26.59%, 2.57 ± 0.43, and 5801.10 ± 631.62 in LPS-stimulated cells to 142.91 ± 20.33% (n = 5, *P* < 0.05 versus the LPS alone-treated group), 1.81 ± 0.16 (n = 3, *P* < 0.05 versus the LPS alone-treated group), and 3839.26 ± 906.27 (n = 6, *P* < 0.05 versus the LPS alone-treated group) in LPS/TSG co-treated cells, respectively. These results suggest that inhibition of induction of pro-inflammatory factors by TSG might contribute to the amelioration of neuronal injury induced by microglia-conditioned medium.

### TSG reduces gene expression of pro-inflammatory factors in LPS-stimulated BV-2 cells

**Figure 4:**
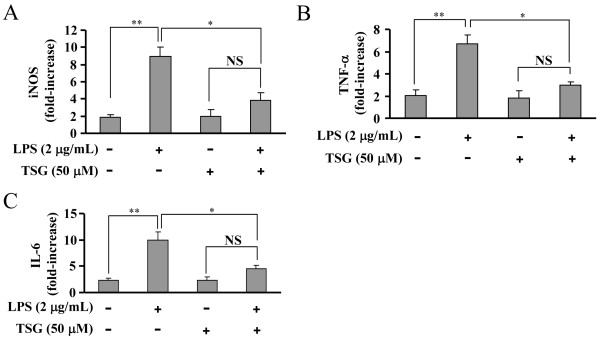
**Role of TSG in mRNA formation of pro-inflammatory factors in BV-2 cells.** Quantitative analysis of the mRNA level of inducible nitric oxide (iNOS) **(A)**, TNF-α **(B)**, and IL-6 **(C)** in cells upon lipolysaccharide (LPS) with or without TSG pretreatment (30 minutes, 50 μM, n = 3, **P < 0.01 versus control; *P < 0.05 versus the LPS alone-treated group). NS, no significance. All data were shown as mean ± standard error.

The reduction of pro-inflammatory factors protein could be due to the suppression of either gene transcription or protein translation. In order to differentiate between the two possibilities, we detected the mRNA level of iNOS, TNF-α, and IL-6 in LPS (2 μg/mL, 24 h)-stimulated BV-2 cells in the absence or presence of TSG by real time-PCR. As shown in Figure 4, LPS induced a robust increase in iNOS, TNF-α, and IL-6 mRNA level. Pre-treatment of cells with TSG (50 μM, 30 minutes) significantly decreased the mRNA level of iNOS (from 8.94 ± 1.06 to 3.83 ± 0.85, n = 3, *P* < 0.05 versus the LPS alone-treated group), TNF-α (from 6.66 ± 0.84 to 2.96 ± 0.33, n = 3, *P* < 0.05 versus the LPS alone-treated group), and IL-6 (from 9.95 ± 1.62 to 4.58 ± 0.65, n = 3, *P* < 0.05 versus the LPS alone-treated group) in LPS-stimulated cells (Figure 4). These data suggest that TSG exerts its inhibitory function likely by reducing gene transcription of pro-inflammatory factors in BV-2 cells.

### TSG does not impact MAPK-IκB-α-NF-κB activation in LPS-stimulated BV-2 cells

**Figure 5:**
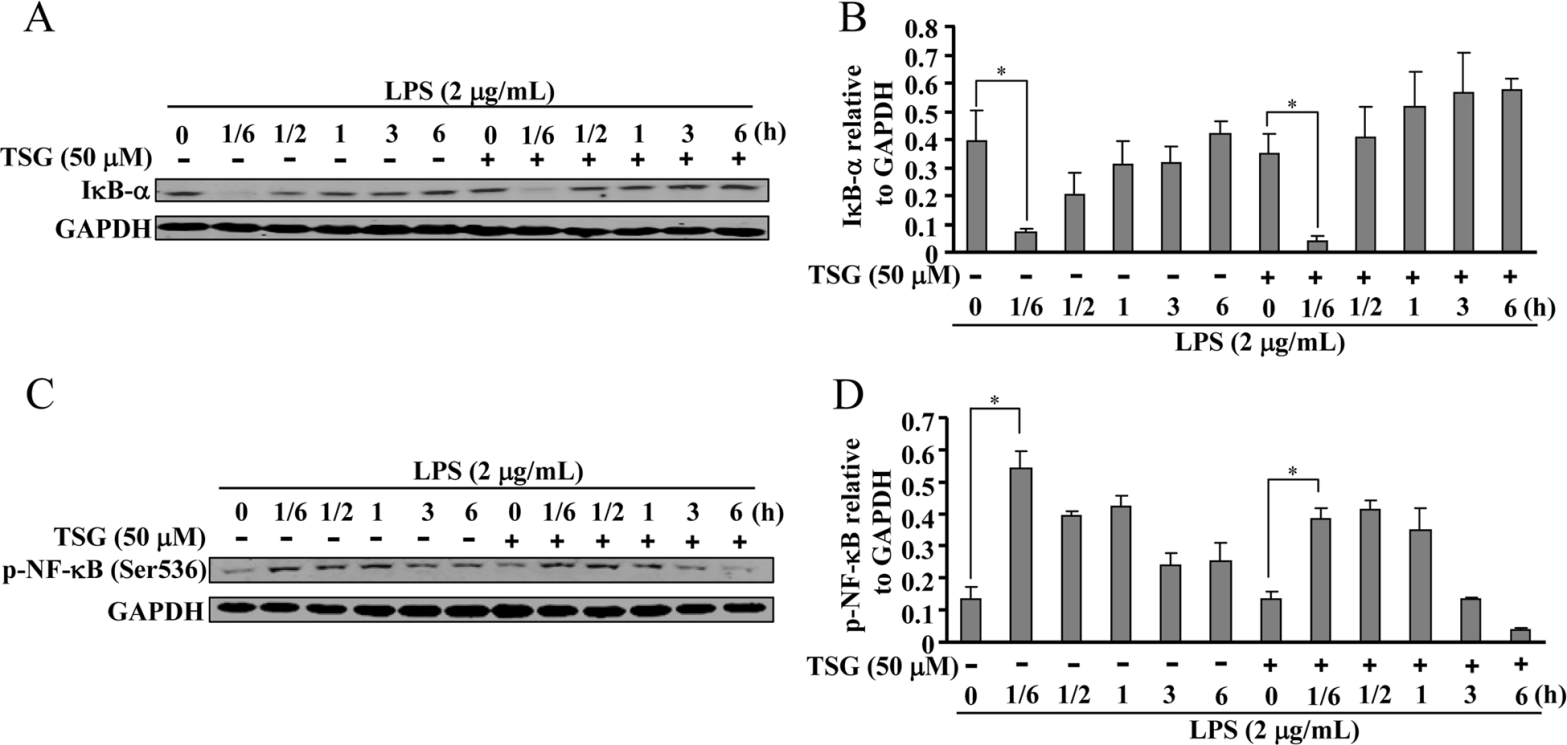
**Effects of TSG on lipopolysaccharide (LPS)-initiated inhibitor of κB α (IκB-α) degradation and nuclear factor κB (NF-κB) phosphorylation in BV-2 cells. (A)** LPS-induced IκB-α degradation was not altered in TSG (50 μM, 30 minutes)-incubated BV-2 cells. **(B)** Quantitative analysis of the effect of TSG on IκB-α degradation in LPS-treated cells (n = 3, *P < 0.05 versus control). **(C)** LPS-induced NF-κB phosphorylation was also not changed by TSG treatment (50 μM, 30 minutes). **(D)** Quantitative analysis of the effect of TSG on NF-κB phosphorylation level in LPS-treated cells (n = 3, *P < 0.05 versus control). All data were shown as mean ± standard error. GAPDH, glyceraldehyde-3-phosphate dehydrogenase.

**Figure 6:**
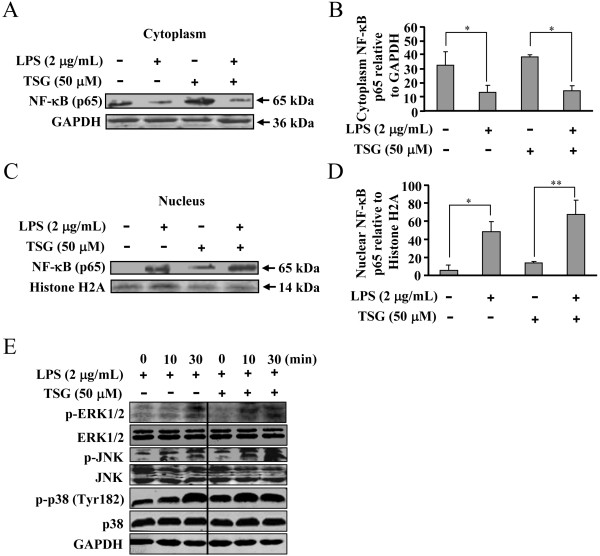
**Effects of TSG on the nuclear translocation of nuclear factor κB (NF-κB) and activation of mitogen-activated protein kinase (MAPK) signals in BV-2 cells. (A)** Effects of TSG on NF-κB expression in the cytoplasm in lipolysaccharide (LPS)/TSG-treated BV-2 cells. **(B)** Quantitative analysis of NF-κB amount in the cytoplasm (n = 3, *P < 0.05 versus control or the TSG alone-treated group). **(C)** Effects of TSG on NF-κB expression in the nucleus in LPS/TSG-treated BV-2 cells. **(D)** Quantitative analysis of the amount of NF-κB in the nucleus (n = 3, *P < 0.05 versus control, **P < 0.01 versus the TSG alone-treated group). **(E)** Representative images showing the effect of TSG (50 μM, 30 minutes) on LPS-induced increase in ERK1/2, JNK, and p38 phosphorylation level in BV-2 cells. Experiments were performed three times independently. All data were shown as mean ± standard error. GAPDH, glyceraldehyde-3-phosphate dehydrogenase.

The IKK-IκB-α-NF-κB signaling pathway is widely accepted to mediate the induction of pro-inflammatory factors in inflammation [17]. Therefore, to explore the mechanism underlying the effect of TSG on gene transcription of pro-inflammatory factors, we measured the change in degradation of IκB-α after TSG treatment in LPS-stimulated BV-2 cells. As shown in Figure 5A and 5B, LPS resulted in a dramatic increase in IκB-α degradation at the time point of 10 minutes, and TSG pretreatment (50 μM, 30 minutes) did not alter this degradation. It has been reported that the full activity of NF-κB needs the increase of NF-κB phosphorylation. We therefore determined whether TSG affects the LPS-triggered NF-κB phosphorylation in BV-2 cells. In accordance with the influence of TSG in IκB-α degradation, TSG treatment (50 μM) failed to reduce the increase in the phosphorylation level of NF-κB p65 at Ser536 in LPS-stimulated BV-2 cells (Figure 5C and D). To initiate gene transcription, active NF-κB must enter nuclei. To investigate whether TSG treatment could influence the nuclear transport of NF-κB, we analyzed the change in NF-κB level in the cytoplasm and nucleus in BV-2 cells stimulated with LPS. As shown in Figure 6A, B, C, and D, NF-κB p65 was present predominantly in the cytoplasm in un-stimulated cells. LPS incubation resulted in NF-κB nuclear translocation, but this translocation was not affected by TSG pretreatment (50 μM, 30 minutes) (Figure 6A, B, C, and D). Because IκB-α degradation and NF-κB activation are reported to be mediated by MAPK signals [18-20], we then checked the influence of TSG in MAPK activation including the phosphorylation of ERK1/2, JNK, and p38. We found that TSG did not suppress the increase in the phosphorylation level of ERK1/2, JNK, and p38 in LPS-stimulated BV-2 cells (Figure 6E). Taken together, our findings indicate that the effect of TSG on induction of pro-inflammatory factors in BV-2 cells was not due to the inhibition of MAPK-IκB-α-NF-κB signals.

### TSG attenuates the binding activity of NF-κB in LPS-stimulated BV-2 cells

**Figure 7:**
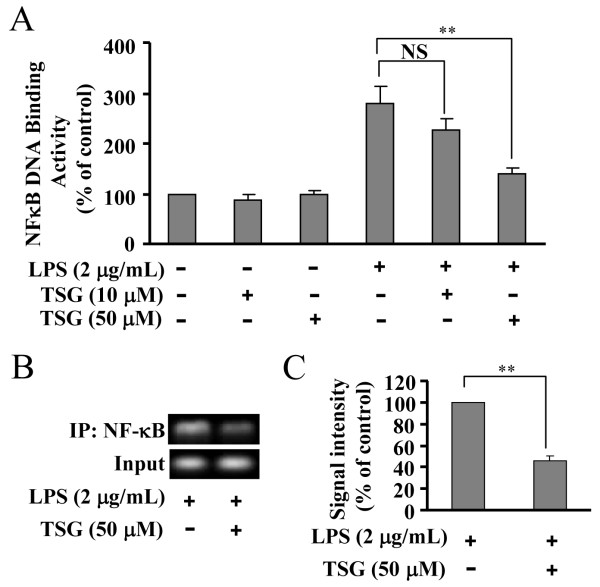
**TSG attenuates the binding of nuclear factor-κB (NF-κB) to its DNA element in BV-2 cells. (A)** Quantitative analysis of the effect of TSG on the binding of NF-κB to its DNA element in BV-2 cells (n = 5, **P < 0.01 versus the lipolysaccharide (LPS) alone-treated group). **(B)** ChIP assays showed that TSG treatment (50 μM, 30 minutes) markedly reduced the binding of NF-κB to the inducible nitric oxide (iNOS) promoter in LPS-treated cells. The chromatin was immunoprecipitated by using the anti-NF-κB p65 antibody. **(C)** Quantitative analysis of the effect of TSG on the binding of NF-κB to the iNOS promoter in BV-2 cells (n = 3, **P < 0.01 versus control). NS, no significance. All data were shown as mean ± standard error.

As TSG did not appear to affect MAPK-IκB-α-NF-κB activation, we then tested a possibility that TSG might inhibit pro-inflammatory factor transcription by directly interfering with NF-κB binding to its DNA element. To explore this possibility, BV-2 cells were stimulated with LPS to activate NF-κB. Binding of active NF-κB with labeled DNA oliogos corresponding to its promoter was monitored in the absence or presence of TSG. As shown in Figure 7A, LPS induced a dramatic increase in NF-κB binding activity in nuclei. This effect was abolished by TSG in a dose-dependent manner. To further confirm this effect, we performed ChIP assays on the iNOS promoter in stimulated cells with or without pretreatment with TSG (50 μM). As shown in Figure 7B and 7C, LPS-elicited NF-κB binding to the iNOS promoter was significantly reduced by TSG pretreatment (50 μM, 30 minutes). These data indicate that TSG primarily interferes with binding of NF-κB to its DNA element in BV-2 microglia.

### TSG reduces the induction of iNOS in LPS-stimulated primary microglia

**Figure 8:**
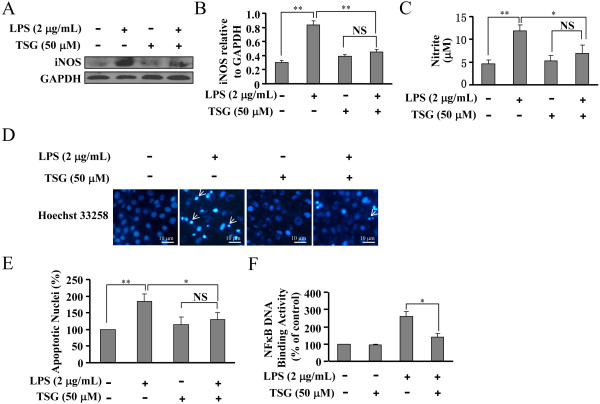
**TSG reduces the induction of inducible nitric oxide (iNOS) in lipolysaccharide (LPS)-stimulated primary microglia and protects the hippocampal neurons from primary microglia conditioned medium-induced cell injury. (A)** Representative images showing that TSG treatment (30 minutes, 50 μM) inhibited iNOS expression in primary microglia. **(B)** Quantitative analysis of the iNOS expression in primary microglia in LPS/TSG-treated cells (n = 5, **P < 0.01 versus control or the LPS alone-treated group). **(C)** Quantitative analysis of NO content in LPS/TSG-treated cells (n = 5, **P < 0.01 versus control; *P < 0.05 versus the LPS alone-treated group). **(D)** TSG treatment (30 minutes, 50 μM) inhibited the increase in apoptotic nuclei in hippocampal neurons induced by primary microglia-derived conditioned medium. **(E)** Quantitative analysis of apoptotic nuclei in LPS/TSG-treated cells (n = 5, **P < 0.01 versus control; *P < 0.05 versus the LPS alone-treated group). **(F)** Quantitative analysis showing that the LPS-induced increase in the binding of nuclear factor -κB (NF-κB) to its DNA element in primary microglia was attenuated by TSG treatment at the concentrations of 10 and 50 μM (n = 5, *P < 0.05 versus the LPS alone-treated group). NS, no significance; GAPDH, glyceraldehyde-3-phosphate dehydrogenase. All data were shown as mean ± standard error.

Finally, we performed experiments to ascertain whether the major findings in BV-2 cells also occur in primary microglia. As expected, pretreatment of primary microglia with TSG (50 μM, 30 minutes) reduced the iNOS expression (Figure 8A and B). Similar to what was observed in BV-2 cells, NO production in LPS-stimulated primary microglia was also reduced by TSG treatment (50 μM, 30 minutes) (Figure 8C). Consequently, TSG reduced the percentage of apoptotic nuclei in hippocampal neurons injured by primary microglia-derived conditioned medium (Figure 8D and E). Moreover, we observed an inhibitory effect of TSG on the binding of NF-κB to its DNA element in the nucleus in LPS-stimulated primary microglia (Figure 8F). Collectively, these data demonstrate that TSG attenuates the inflammatory response in primary microglia by suppressing the DNA binding activity of NF-κB.

## Discussion

The endotoxin- or pathogen-mediated induction of pro-inflammatory factors in microglia are implicated in pathophysiological processes of neurotoxicity [33-35]. Numerous anti-oxidative molecules have been shown to protect neurons from cell toxicity in inflammatory disorders by attenuating the production of pro-inflammatory factors such as NO, TNF-α, and IL-6 in microglia [21,22,36]. TSG, an active component of the rhizome extract from *Polygonummultiflorum*, exhibits its function through anti-inflammation [24], anti-apoptosis [26], and anti-oxidation [37]. In this study, we found that TSG impairs LPS-mediated inflammatory response in microglia. This effect was exemplified by the decrease in the production of pro-inflammatory factors as well as the DNA binding activity of NF-κB in LPS/TSG-stimulated microglia. This finding underscores the importance of TSG in regulation of inflammation, and extends the role of TSG beyond a cardiovascular protective molecule to a modulator of microglia activation. Recently, interesting work reported that TSG prevents the overexpression of α-synuclein in APPV717I transgenic mice with Alzheimer’s disease (AD), strongly showing a potential role of TSG in prevention or treatment of AD [38]. Together with the fact that microglia are believed to mediate the development of AD [39,40] and that the damage of neurons in neurodegenerative disorders is usually secondary to microglia activation [41,42], we believe that our data may provide evidence to clarify how TSG exerts its protective effect in AD. *In vivo* experiments designed to investigate the role and mechanism of TSG in different neurodegenerative disorders in the CNS are in progress.

The functional consequence of microglia activation depends on the induction and release of pro-inflammatory factors. The appropriate amount of pro-inflammatory factor is indispensable for various physiological processes, such as neuronal protection [14,15] and synaptic plasticity [31]. However, overwhelmingly generated pro-inflammatory factors can act as neurotoxins and cause neuronal injury [5,6,43,44]. Strategies to inhibit the excessive production of pro-inflammatory factors are crucial to attenuate the neurotoxicity induced by inflammatory events. In the present study, we showed that TSG reduces the content of NO, TNF-α, and IL-6, and this reduction protects the hippocampal neurons from microglia-conditioned medium-induced cell injury. This finding about the role of TSG in induction of pro-inflammatory factors in microglia and protection of hippocampal neurons from inflammatory stimulation provides a new insight into the pharmacological role of TSG in inflammatory disorders.

Blockade of gene transcription in stimulated inflammatory cells is often due to one or multiple interruptions in the signaling transduction from the stimuli to the corresponding transcriptional cytokines. In LPS signals, the MAPKs-IKK-IκB-α-NF-κB pathway is downstream of LPS signaling transduction [15,34]. IκB-α is phosphorylated by IKK, which then leads to the degradation of IκB-α and the translocation of NF-κB from the cytoplasm to nucleus. However, we found that although TSG inhibited pro-inflammatory factor gene-transcription in microglia, it failed to affect LPS-induced IκB-α degradation and NF-κB phosphorylation or nuclear translocation. Neither did we observe any significant effect on the LPS-induced increase in ERK1/2, JNK, or the p38 phosphorylation levels. It indicates that TSG might affect the inflammatory response in microglia by a mechanism downstream of the nuclear translocation of NF-κB. In fact, our results showed that the increase in DNA binding activity of NF-κB in LPS-stimulated microglia was remarkably suppressed by TSG. Moreover, the reduction in active NF-κB from binding to the iNOS promoter in the ChIP assay further confirmed the function of TSG. In general, these data provide a plausible explanation why the gene transcription of pro-inflammatory factors is inhibited, despite the fact that activation of MAPK-IκB-α-NF-κB signals is not perturbed in TSG/LPS co-treated cells. However, as we did not check the influence of TSG in the binding of NF-κB to TNF-α and IL-6 promoters, to some extent these conclusions limit our observations for the role of TSG in inflammatory response in microglia. Some previous studies associated with the interaction between NF-κB and TNF-α and IL-6 promoters might give us evidence to offset the limitation. For example, a 120-bp TNF-α promoter was found to possess a binding site for NF-κB [45], and the promoter region of the IL-6 gene was confirmed to have a putative NF-κB binding site [46].

It is worth noting that our above observations in microglia are inconsistent with the *in vitro* oxygen glucose deprivation/reperfusion (OGD/R)-stimulated neurons where the TSG-mediated reduction in iNOS expression and brain infarct volume is mediated by the inhibition of nuclear translocation of NF-κB [26]. However, because there are large differences between *in vitro* and *in vivo* micro-environments and the damage of neurons in the *in vivo* conditions usually occurs following excessive activation of microglia, we tend to suppose that the TSG-mediated reduction in infarct volume after brain ischemia might be mediated by the attenuation of inflammatory response in microglia with a mechanism that is different from that in neurons. That is, TSG may protect the brain tissues from ischemia by impairing the DNA binding activity of NF-κB. However, how this mechanism works in microglia is not clear. We notice that though resveratrol, a polyphenolicphytoalexin found in grapes, fruits, and root extracts of the weed *Polygonumcuspidatum*[47], has similar characteristics in structure to TSG [48,49], it exhibits distinct mechanisms in regulation of inflammatory response in microglia. For example, resveratrol was reported to decrease the production of pro-inflammatory factors primarily by impairing the phosphorylation and nuclear translocation of NF-κB [49,50]. This difference, combined with the highly similar structure of TSG and resveratrol, might help us to further clarify the exact mechanism of TSG in inflammatory response.

In summary, this study identifies a role for TSG in the induction of pro-inflammatory factors in microglia by a mechanism that is independent of MAPK activation, IκB-α degradation, and NF-κB phosphorylation/nuclear translocation, but probably relies on the repression of NF-κB binding activity.

## Abbreviations

AD: Alzheimer’s disease; ANOVA: Analysis of variance; Bp: Base pairs; ChIP: Chromatin immunoprecipitation; CNS: Central nervous system; Ct: Cycle threshold; DMEM: Dulbecco’s modified Eagle’s medium; eNOS: Endothelial nitric oxide synthase; FBS: Fetal bovine serum; GAPDH: Glyceraldehyde-3-phosphate dehydrogenase; IL: Interleukin; iNOS: Inducible nitric oxide synthase; IκB-α: Inhibitor of κB-α; IKK: Inhibitor of κB kinase; LDH: Lactate dehydrogenase; LPS: Lipopolysaccharide; MAPK: Mitogen-activated protein kinase; NF-κB: Nuclear factor-κB; NO: Nitric oxide; NOS: Nitric oxide synthase; nNOS: Neuronal nitric oxide synthase; PBS: Phosphate-buffered saline; PCR: Polymerase chain reaction; SE: Standard error; TBST: Tris-buffered saline Tween-20; TNF-α: Tumor necrosis factor-α; TSG: 2,3,4’,5-tetrahydroxystilbene 2-O-β-D-glucoside.

## Competing interests

The authors declare that they have no competing interests.

## Authors’ contributions

CH participated in the design of this study. CH and YW performed the cell culture, western blot, statistical analysis in the whole research. YW carried out the real time-PCR assay. JW carried out the NO assay. WY participated in the design of this study and carried out the LDH and MTT assay. CH carried out Hoechst 33258 staining. XC and WY participated in the design of this study. YW carried out the NF-κB binding and ChIP assays. WZ participated in the design of this study and proofread the whole manuscript. All authors read and approved the final manuscript.

## References

[B1] Graeber MB Changing face of microglia Science 2010 330 783 788 10.1126/science.1190929 21051630

[B2] Halleskog C Mulder J Dahlström J Mackie K Hortobágyi T Tanila H Kumar Puli L Färber K Harkany T Schulte G WNT signaling in activated microglia is proinflammatory Glia 2011 59 119 131 10.1002/glia.21081 20967887 PMC3064522

[B3] Levy A Bercovich-Kinori A Alexandrovich AG Tsenter J Trembovler V Lund FE Shohami E Stein R Mayo L CD38 facilitates recovery from traumatic brain injury J Neurotrauma 2009 26 1521 1531 10.1089/neu.2008.0746 19257806 PMC2864472

[B4] Calabrese V Mancuso C Calvani M Rizzarelli E Butterfield DA Stella AM Nitric oxide in the central nervous system: neuroprotection versus neurotoxicity Nat Rev Neurosci 2007 8 766 775 10.1038/nrn2214 17882254

[B5] Takeuchi H Jin S Wang J Zhang G Kawanokuchi J Kuno R Sonobe Y Mizuno T Suzumura A Tumor necrosis factor-alpha induces neurotoxicity via glutamate release from hemichannels of activated microglia in an autocrine manner J Biol Chem 2006 281 21362 21368 10.1074/jbc.M600504200 16720574

[B6] Ma TC Zhu XZ Neurotoxic effects of interleukin-6 and sodium nitroprusside on cultured rat hippocampal neurons Arzneimittelforschung 2000 50 512 514 10918941 10.1055/s-0031-1300239

[B7] Andrew PJ Mayer B Enzymatic function of nitric oxide synthases Cardiovasc Res 1999 43 521 531 10.1016/S0008-6363(99)00115-7 10690324

[B8] Liu S Premont RT Rockey DC G-protein-coupled receptor kinase interactor-1 (GIT1) is a new endothelial nitric-oxide synthase (eNOS) interactor with functional effects on vascular homeostasis J Biol Chem 2012 287 12309 12320 10.1074/jbc.M111.320465 22294688 PMC3320980

[B9] Gallo EF Iadecola C Neuronal nitric oxide contributes to neuroplasticity-associated protein expression through cGMP, protein kinase G, and extracellular signal-regulated kinase J Neurosci 2011 31 6947 6955 10.1523/JNEUROSCI.0374-11.2011 21562256 PMC3110776

[B10] MacMicking JD Nathan C Hom G Chartrain N Fletcher DS Trumbauer M Stevens K Xie QW Sokol K Hutchinson N Altered responses to bacterial infection and endotoxic shock in mice lacking inducible nitric oxide synthase Cell 1995 81 641 650 10.1016/0092-8674(95)90085-3 7538909

[B11] Griffith OW Steuhr DJ Nitric oxide synthases: properties and catalytic mechanism Annu Rev Physiol 1995 57 707 736 10.1146/annurev.ph.57.030195.003423 7539994

[B12] Singh S Das T Ravindran A Chaturvedi RK Shukla Y Agarwal AK Dikshit M Involvement of nitric oxide in neurodegeneration: a study on the experimental models of Parkinson’s disease Redox Rep 2005 10 103 109 10.1179/135100005X38842 15949131

[B13] Smith KJ Lassmann H The role of nitric oxide in multiple sclerosis Lancet Neurol 2002 1 232 241 10.1016/S1474-4422(02)00102-3 12849456

[B14] Saha RN Ghosh A Palencia CA Fung YK Dudek SM Pahan K TNF-alpha preconditioning protects neurons via neuron-specific up-regulation of CREB-binding protein J Immunol 2009 183 2068 2078 10.4049/jimmunol.0801892 19596989 PMC2724010

[B15] Tran HY Shin EJ Saito K Nguyen XK Chung YH Jeong JH Bach JH Park DH Yamada K Nabeshima T Yoneda Y Kim HC Protective potential of IL-6 against trimethyltin-induced neurotoxicity in vivo Free Radic Biol Med 2012 52 1159 1174 10.1016/j.freeradbiomed.2011.12.008 22245015

[B16] Kleinert H Pautz A Linker K Schawz PM Regulation of the expression of inducible nitric oxide synthase Eur J Pharmacol 2004 500 255 266 10.1016/j.ejphar.2004.07.030 15464038

[B17] Hayden MS Ghosh S Signaling to NF-κB Genes Dev 2004 18 2195 2224 10.1101/gad.1228704 15371334

[B18] Jang SI Kim HJ Kim YJ Jeong SI You YO TanshinoneIIA inhibits LPS-induced NF-kappaB activation in RAW 264.7 cells: possible involvement of the NIK-IKK, ERK1/2, p38 and JNK pathways Eur J Pharmacol 2006 542 1 7 10.1016/j.ejphar.2006.04.044 16797002

[B19] Tang G Minemoto Y Dibling B Purcell NH Li Z Karin M Lin A Inhibition of JNK activation through NF-kappaB target genes Nature 2001 414 313 317 10.1038/35104568 11713531

[B20] Chio CC Chang YH Hsu YW Chi KH Lin WW PKA-dependent activation of PKC, p38 MAPK and IKK in macrophage: implication in the induction of inducible nitric oxide synthase and interleukin-6 by dibutyryl cAMP Cell Signal 2004 16 565 575 10.1016/j.cellsig.2003.10.003 14751542

[B21] Lee CJ Lee SS Chen SC Ho FM Lin WW Oregonin inhibits lipopolysaccharide-induced iNOS gene transcription and upregulates HO-1 expression in macrophages and microglia Br J Pharmacol 2005 146 378 388 10.1038/sj.bjp.0706336 16025135 PMC1576284

[B22] Karlstetter M Lippe E Walczak Y Moehle C Aslanidis A Mirza M Langmann T Curcumin is a potent modulator of microglial gene expression and migration J Neuroinflammation 2011 8 125 10.1186/1742-2094-8-125 21958395 PMC3192695

[B23] Wang X Zhao L Han T Chen S Wang J Protective effects of 2,3,5,4’-tetrahydroxystilbene-2-O-beta-d-glucoside, an active component of PolygonummultiflorumThunb, on experimental colitis in mice Eur J Pharmacol 2008 578 339 348 10.1016/j.ejphar.2007.09.013 17963744

[B24] Zhang YZ Shen JF Xu JY Xiao JH Wang JL Inhibitory effects of 2,3,5,4’-tetrahydroxystilbene-2-O-beta-D-glucoside on experimental inflammation and cyclooxygenase 2 activity J Asian Nat Prod Res 2007 9 355 363 10.1080/10286020600727772 17613621

[B25] Zhang W Xu XL Wang YQ Wang CH Zhu WZ Effects of 2,3,4’,5-tetrahydroxystilbene 2-O-beta-D-glucoside on vascular endothelial dysfunction in atherogenic-diet rats Planta Med 2009 75 1209 1214 10.1055/s-0029-1185540 19350477

[B26] Wang T Gu J Wu PF Wang F Xiong Z Yang YJ Wu WN Dong LD Chen JG Protection by tetrahydroxystilbene glucoside against cerebral ischemia: involvement of JNK, SIRT1, and NF-kappaB pathways and inhibition of intracellular ROS/RNS generation Free Radic Biol Med 2009 47 229 240 10.1016/j.freeradbiomed.2009.02.027 19272442

[B27] Paakkari I Lindsberg P Nitric oxide in the central nervous system Ann Med 1995 27 369 377 10.3109/07853899509002590 7546627

[B28] Saha RN Pahan K Regulation of inducible nitric oxide synthase gene in glial cells Antioxid Redox Signal 2006 8 929 947 10.1089/ars.2006.8.929 16771683 PMC1963415

[B29] Huang C Hu ZL Wu WN Yu DF Xiong QJ Song JR Shu Q Fu H Wang F Chen JG Existence and distinction of acid-evoked currents in rat astrocytes Glia 2010 58 1415 1424 20549751 10.1002/glia.21017

[B30] Liu Y Kintner DB Chanana V Algharabli J Chen X Gao Y Chen J Ferrazzano P Olson JK Sun D Activation of microglia depends on Na^+^/H^+^exchange-mediated H^+^homeostasis J Neurosci 2010 30 15210 15220 10.1523/JNEUROSCI.3950-10.2010 21068326 PMC3010222

[B31] Huang C Wang J Chen Z Wang Y Zhang W 2-Phenylethynesulfonamide prevents induction of Pro-inflammatory factors and attenuates LPS-induced liver injury by targeting NHE1-Hsp70 complex in mice PLoS One 2013 8 e67582 10.1371/journal.pone.0067582 23805318 PMC3689707

[B32] Luo S Wang T Qin H Lei H Xia Y Obligatory role of heat shock protein 90 in iNOS induction Am J Physiol Cell Physiol 2011 301 C227 C233 10.1152/ajpcell.00493.2010 21430289 PMC3129818

[B33] Moehle MS Webber PJ Tse T Sukar N Standaert DG DeSilva TM Cowell RM West AB LRRK2 inhibition attenuates microglial inflammatory responses J Neurosci 2012 32 1602 1611 10.1523/JNEUROSCI.5601-11.2012 22302802 PMC3532034

[B34] Starossom SC Mascanfroni ID Imitola J Cao L Raddassi K Hernandez SF Bassil R Croci DO Cerliani JP Delacour D Wang Y Elyaman W Khoury SJ Rabinovich GA Galectin-1 deactivates classically activated microglia and protects from inflammation-induced neurodegeneration Immunity 2012 37 249 263 10.1016/j.immuni.2012.05.023 22884314 PMC3428471

[B35] Mir M Tolosa L Asensio VJ Lladó J Olmos G Complementary roles of tumor necrosis factor alpha and interferon gamma in inducible microglial nitric oxide generation J Neuroimmunol 2008 204 101 109 10.1016/j.jneuroim.2008.07.002 18703234

[B36] Lu J Wu DM Zheng YL Hu B Zhang ZF Shan Q Zheng ZH Liu CM Wang YJ Quercetin activates AMP-activated protein kinase by reducing PP2C expression protecting old mouse brain against high cholesterol-induced neurotoxicity J Pathol 2010 222 199 212 10.1002/path.2754 20690163

[B37] Zhang SH Wang WQ Wang JL Protective effect of tetrahydroxystilbene glucoside on cardiotoxicity induced by doxorubicin in vitro and in vivo Acta Pharmacol Sin 2009 30 1479 1487 10.1038/aps.2009.144 19890356 PMC4003005

[B38] Zhang L Yu S Zhang R Xing Y Li Y Li L Tetrahydroxystilbene glucoside antagonizes age-related α-synuclein overexpression in the hippocampus of APP transgenic mouse model of Alzheimer’s disease Restor Neurol Neurosci 2013 31 41 52 23160059 10.3233/RNN-120260

[B39] Orre M Kamphuis W Dooves S Kooijman L Chan ET Kirk CJ Dimayuga Smith V Koot S Mamber C Jansen AH Ovaa H Hol EM Reactive glia show increased immunoproteasome activity in Alzheimer’s disease Brain 2013 136 1415 1431 10.1093/brain/awt083 23604491

[B40] Solito E Sastre M Microglia function in Alzheimer’s disease Front Pharmacol 2012 3 14 22363284 10.3389/fphar.2012.00014 PMC3277080

[B41] Qu WS Tian DS Guo ZB Fang J Zhang Q Yu ZY Xie MJ Zhang HQ Lü JG Wang W Inhibition of EGFR/MAPK signaling reduces microglial inflammatory response and the associated secondary damage in rats after spinal cord injury J Neuroinflammation 2012 23 178 22824323 10.1186/1742-2094-9-178 PMC3418570

[B42] Zhang QG Laird MD Han D Nguyen K Scott E Dong Y Dhandapani KM Brann DW Critical role of NADPH oxidase in neuronal oxidative damage and microglia activation following traumatic brain injury PLoS One 2012 7 e34504 10.1371/journal.pone.0034504 22485176 PMC3317633

[B43] Förstermann U Sessa WC Nitric oxide synthases: regulation and function Eur Heart J 2012 33 829 837 10.1093/eurheartj/ehr304 21890489 PMC3345541

[B44] Volbracht C Chua BT Ng CP Bahr BA Hong W Li P The critical role of calpain versus caspase activation in excitotoxic injury induced by nitric oxide J Neurochem 2005 93 1280 1292 10.1111/j.1471-4159.2005.03122.x 15934947

[B45] Liu H Sidiropoulos P Song G Pagliari LJ Birrer MJ Stein B Anrather J Pope RM TNF-alpha gene expression in macrophages: regulation by NF-kappa B is independent of c-Jun or C/EBP beta J Immunol 2000 164 4277 4285 10754326 10.4049/jimmunol.164.8.4277

[B46] Libermann TA Baltimore D Activation of interleukin-6 gene expression through the NF-kappa B transcription factor Mol Cell Biol 1990 10 2327 2334 2183031 10.1128/mcb.10.5.2327-2334.1990 PMC360580

[B47] Soleas GJ Diamandis EP Goldberg DM Resveratrol: a molecule whose time has come? And gone? Clin Biochem 1997 30 91 10.1016/S0009-9120(96)00155-5 9127691

[B48] Han X Ling S Gan W Sun L Duan J Xu JW 2,3,5,4’-tetrahydroxystilbene-2-O-β-d-glucoside ameliorates vascular senescence and improves blood flow involving a mechanism of p53 deacetylation Atherosclerosis 2012 225 76 82 10.1016/j.atherosclerosis.2012.08.011 22981429

[B49] Capiralla H Vingtdeux V Zhao H Sankowski R Al-Abed Y Davies P Marambaud P Resveratrol mitigates lipopolysaccharide- and Aβ-mediated microglial inflammation by inhibiting the TLR4/NF-κB/STAT signaling cascade J Neurochem 2012 120 461 472 10.1111/j.1471-4159.2011.07594.x 22118570 PMC3253186

[B50] Yi CO Jeon BT Shin HJ Jeong EA Chang KC Lee JE Lee DH Kim HJ Kang SS Cho GJ Choi WS Roh GS Resveratrol activates AMPK and suppresses LPS-induced NF-κB-dependent COX-2 activation in RAW 264.7 macrophage cells Anat Cell Biol 2011 44 194 203 10.5115/acb.2011.44.3.194 22025971 PMC3195823

